# Learning the effective order of a hypergraph dynamical system

**DOI:** 10.1126/sciadv.adh4053

**Published:** 2024-05-08

**Authors:** Leonie Neuhäuser, Michael Scholkemper, Francesco Tudisco, Michael T. Schaub

**Affiliations:** ^1^ RWTH Aachen University, Aachen, Germany.; ^2^ GSSI Gran Sasso Science Institute, L’Aquila, Italy.; ^3^ School of Mathematics and Maxwell Institute, University of Edinburgh, Peter Guthrie Tait Road, EH9 3FD, Edinburgh, UK.

## Abstract

Dynamical systems on hypergraphs can display a rich set of behaviors not observable for systems with pairwise interactions. Given a distributed dynamical system with a putative hypergraph structure, an interesting question is thus how much of this hypergraph structure is actually necessary to faithfully replicate the observed dynamical behavior. To answer this question, we propose a method to determine the minimum order of a hypergraph necessary to approximate the corresponding dynamics accurately. Specifically, we develop a mathematical framework that allows us to determine this order when the type of dynamics is known. We use these ideas in conjunction with a hypergraph neural network to directly learn the dynamics itself and the resulting order of the hypergraph from both synthetic and real datasets consisting of observed system trajectories.

## Teaser

A method is introduced to learn what higher-order interactions are needed to accurately represent a hypergraph dynamical system.

## INTRODUCTION

Dynamical processes on hypergraphs (*1*, *2*) have recently received substantial attention. Examples include synchronization (*3*, *4*), consensus dynamics (*5*–*7*), epidemic spread (*8*–*10*), random walks (*11*, *12*), label propagation (*13*–*15*), and social contagion (*16*, *17*). Generally, it has been found that, for dynamical systems supported on hypergraphs instead of graphs, important characteristics of the dynamics can change. For example, contagion processes on hypergraphs can have different epidemic thresholds for the outbreak of an epidemic (*9*, *10*), or group reinforcement effects can strongly alter the outcome of opinion formation on hypergraphs (*17*–*19*). Hypergraphs have also gained interest as extensions of graph neural networks and been used for a variety of applications ranging from three-dimensional shape retrieval (*20*) and pose estimation (*21*) in computer vision to group recommendation tasks (*22*) or medical applications such as cancer tissue classification (*23*).

Although both aspects often appear lumped together in the mathematical equations describing a dynamical system on a hypergraph, from a modeling perspective, it is important to distinguish between (i) the topological relations constraining the possible interactions in a system (as encoded in a hypergraph) and (ii) the model of the local multi-way dynamics occurring on each hyperedge (e.g., epidemic spread, diffusion, and synchronization). It is the interplay of both aspects that leads to the (possible) emergence of higher-order effects (*2*, *18*, *24*).

For instance, if we consider a linear dynamics on a hypergraph, then we cannot expect any higher-order effects to emerge. As any linear map between finite-dimensional spaces can be represented by a matrix, for linear dynamics, we may always find an equivalent graph-based, pairwise interaction dynamics that models the system exactly, by identifying the matrix with an effective weighted graph (*5*, *6*). In practice, this means that it is possible to use a graph instead of a hypergraph-based dynamical system, as long as linear dynamics are considered. Similarly, it has been shown that various formulations of semi-supervised and unsupervised spectral clustering problems on hypergraphs lead to an effective graph-theoretic problem (*25*–*27*).

More generally, we can envision that, depending on the local dynamics, we will be able to rewrite a hypergraph dynamical system on a hypergraph of general order *k* as a dynamics occurring on a hypergraph with hyperedges of order at most *p* ≤ *k*. Such a simplification to lower-order relations is relevant as the use of hypergraphs presents several challenges: most prominently, because the number of hyperedges can grow combinatorially with the number of nodes, the use of hypergraph models can be computationally very expensive. This is particularly relevant for large-scale systems.

Yet, in practice, we typically know neither the exact set of relations between the entities nor the analytical form of the local interaction dynamics but are only given observational data, e.g., in the form of trajectories. For example, we could observe the spread of an epidemic in a population as trajectories of the individuals and their contact patterns, represented by a hypergraph. Another example is the measurement of the abundance of species and their interactions within an ecological system. Many different approaches have been explored to approximate such time-series data. For example, it has recently been proposed to treat neural networks as models equipped with a continuum of layers (*28*). This view allows a reformulation of the forward pass of a neural network as the solution of an initial value problem of an ordinary differential equation (ODE). Deep learning architectures that use this reinterpretation of the forward pass are called neural ODEs and are useful for building continuous-time time-series models. Neural ODEs have also been recently generalized to graphs (*29*, *30*). Discovering the equations governing a dynamical system from measurements is an important problem, tackled by a large body of literature (*31*–*34*). The main challenge here is to find a model that is complex enough to describe the existing data but not so complex as to introduce overfitting.

For a dynamical system on a hypergraph, there are many possible ways to abstract an observed distributed dynamics. For instance, we may model it as emerging from a simple local dynamics on a rather complex hypergraph. Alternatively, we may consider a more complicated local dynamics interacting via a more constrained set of relations between entities. This raises the question of whether we should include the complexity of our model in the topology of the interactions or in the model of the dynamics: What multi-way relations do we need to encode in our model in practice?

Here, we consider a broad family of hypergraph dynamical systems, and we introduce the concepts of the topological and the dynamical order of the system, which measure how complex the dynamics are according to different perspectives. On the basis of the combination of topological and dynamical order, we can determine the effective order of the hypergraph dynamical system, which is given by the minimum order of a hypergraph necessary to exactly represent the corresponding dynamics. In particular, we propose a framework that allows us to derive the dynamical and the effective order when the functional form of the dynamics is given. Further, to infer the effective dynamical order from empirical data, we propose a hypergraph neural network architecture that allows us to learn the hypergraph dynamics and the resulting effective order directly from data, which we test on both synthetic and real datasets. In conclusion, we present an effective method to reduce the complexity of a family of hypergraph dynamical systems and to learn their representation from data.

## REDUCIBILITY OF HYPERGRAPH DYNAMICAL SYSTEMS

To illustrate our ideas of the dynamical and effective order of a dynamical system on a hypergraph, let us start with a Kuramoto-type dynamics (*35*) on a hypergraph as a concrete example. Kuramoto oscillator dynamics have been applied to various synchronization phenomena of phase oscillators (*36*), ranging from power networks (*37*) to brain activity (*38*). Several works have been working on its generalization to simplicial complexes (*3*, *4*) and hypergraphs (*39*). We will compare two different formulations of Kuramoto dynamics on a hypergraph here.

Consider a hypergraph *ℋ* consisting of a set 𝒱 = {1,2⋯, *N*} of *N* nodes and a set ε = {*E*_1_, *E*_2_, …, *E_M_*} of *M* hyperedges. Each hyperedge *E*_α_ is a subset of the nodes, i.e., *E*_α_ ⊆ *V* for all α = 1,2, …, *M*. Each hyperedge may have a different cardinality ∣*E*_α_∣. The topological order of a hypergraph is defined as follows:

**Definition 1.** (Topological order) The topological order of a hypergraph *H* is given by the cardinality of its largest hyperedge *k* = max_α_ ∣*E*_α_∣.

Let *x*(*t*) ∈ ℝ*^N^* be the vector of dynamical state variables of the nodes at time *t*. For simplicity, we will suppress the time dependency and write *x_i_* = *x_i_*(*t*) for the *i*th component of the node state vector in the following.

There are several ways in which the well-known Kuramoto dynamics can be extended to hypergraphs. First, inspired by Adhikari *et al.* (*39*), we can write the time evolution of node *i* as

$$x_{i}=\sum_{\alpha :i\in E_{\alpha }}\;\text{sin}[\sum_{j\in E_{\alpha }}\;\left(x_{j}-x_{i}\right)]$$ (1)

Because the nonlinear sine function acts within each entire hyperedge, for general node states, *x_i_* it is not clear how to further reduce the system to a dynamical system on a lower-order hypergraph.

An alternative formulation for Kuramoto oscillator dynamics is

$$x_{i}=\sum_{\alpha :i\in E_{\alpha }}\;\sum_{j\in E_{\alpha }}\;\text{sin}\left(x_{j}-x_{i}\right)=\sum_{j=1}^{N}\;A_{\mathrm{ij}}\text{sin}\left(x_{j}-x_{i}\right)$$ (2)

where, within each hyperedge, every pair of nodes interacts and

$$A_{\mathrm{ij}}=\left\{ \begin{array}{cc} 1 & \text{if}\;i\in E_{\alpha }\;\text{and}\;j\in E_{\alpha }\\ 0 & \text{else} \end{array}\right.$$ (3)

Hence, although we are dealing here with a nonlinear dynamics on a hypergraph, the dynamics in each hyperedge is pairwise, and we can reduce it to a pairwise network dynamics: The hypergraph topology simply scales the system by *A*.

Overall, our example of Kuramoto oscillator dynamics shows that whether the hypergraph can be projected onto a lower-order system depends on the form of the dynamics supported on each hyperedge: The dynamics is reducible if the dynamics on the hyperedges can be rewritten as a linear combination of lower-order functions. More generally, for certain functional forms, the nonlinear dynamics can always be reduced to a lower-order hypergraph system. In section S1, we show the linear-like properties that dynamics must have to be reducible to a network dynamical system.

In the following, we formalize this form of dynamical reduction by distinguishing between the topological order of a hypergraph and the dynamical order of a dynamics. Combining topology and dynamics yields a hypergraph dynamical system, whose effective order cannot be larger than the minimum of these two orders.

### Dynamical order and effective order

Here, we introduce a framework to separate the impact of topology and dynamics for a broad family of hypergraph dynamical systems. To this end, we group the edges of a hypergraph *H* by size, so that its edge set is given by ℰ = ℰ_2_ ∪ ⋯ ∪ ℰ*_k_* where *E_d_* denotes the set of all hyperedges of size *d*. We refer to the values in the state vector *x*(*t*) of nodes in the hyperedge *E* at time *t* excluding node *i* with {{*x_j_*(*t*) ∣ *j* ∈ *E*, *j* ≠ *i*}}. This is a multiset, which we denote by double braces, i.e., a set where elements may occur multiple times, because the node may have the same values. Formally, we now define the family of hypergraph dynamical systems subject of our investigation.

**Definition 2.** (Hypergraph dynamical system) Consider a hypergraph *H* with topological order *k*. A hypergraph dynamical system is defined by means of a family of multivariate update functions (*f_d_*)_*d* ∈ 1…*k*_, where each function *f_d_*(*y*_1_, …, *y_d_*) acts on *d* variables, and *f_d_* is symmetric in its last *d* − 1 arguments, i.e., any permutation of the last *d* − 1 variables does not change the function. Note that, with a slight abuse of notation, for any such function it holds

$$f_{d}\left(y_{1},y_{2},\ldots ,y_{d}\right)=f_{d}\left(y_{1},\{\{y_{2},\ldots ,y_{d}\}\}\right)$$

if we implicitly agree that the multiset here coincides with any arbitrarily ordered sequence of its elements. These update functions *f_d_* describe the local dynamics on each hyperedge and, for each node *i*, the hypergraph dynamical system on *H* is defined by the update equations

$${\dot{x}}_{i}=f_{1}\left(x_{i}\right)+\underset{\text{topology}}{\underbrace{\sum_{d=2}^{k}\;\sum_{E\in {ℰ}_{d}:i\in E}}}\underset{\text{dynamics}}{\underbrace{f_{d}\left(x_{i},\{\{x_{j}|j\in E,j\neq i\}\}\right)}}$$ (4)

for *i* = 1,…, *N*.

Note that Eq. 4 corresponds to a composition of three operations: First, the state vector is lifted to a higher-dimensional space corresponding to all the hyperedges; then, the mappings *f_d_* are applied to the lifted state space; last, following this generally nonlinear update step, the states are projected back onto the nodes via the transposed lifting map. With this procedure, Eq. 4 separates the hypergraph topology from the dynamics on the edges: For each size *d*, a set of hyperedges defines the topological coupling of entities within the system, and the update functions *f_d_* act on the *d* state variables associated to the nodes within each of these hyperedges. This interpretation is formalized and detailed in Methods.

As we are primary interested in interacting dynamics, for simplicity, we will set *f*_1_ = 0 in the following, although all our arguments can be naturally extended to the nonzero case. It is important to remark that the above functional form is fairly general: The only restriction that we make here is that, within each hyperedge, neighboring nodes act dynamically identical, i.e., the last *d* − 1 entries in *f_d_* are symmetric. This assumption may be further relaxed if we are willing to assign different class labels to different nodes a priori, but we consider here the case in which all nodes act alike. Crucially, the additive expansion that we consider here will always be well-defined in this context, although it may become trivial if only the last term describing the interaction between all nodes within an hyperedge remains.

Many commonly used hypergraph dynamical processes considered in the recent literature are directly expressible in the above form, see, e.g., (*6*, *10*, *11*, *14*, *17*). For example, the dynamics of the Kuramoto oscillator model on hyperedges in Eq. 1 can be written as above if we define *f*_1_ = 0 and

$$f_{d}\left(y_{1},\ldots ,y_{d}\right)=\text{sin}[\sum_{i=1}^{d}\;\left(y_{i}-y_{1}\right)]\;\text{for}\;d=2,\ldots ,k$$

We now introduce the dynamical order of such a hypergraph dynamical system, determined by the form of the update functions *f_d_*.

**Definition 3.** (Dynamical order) Consider a hypergraph dynamical system. Each update function *f_d_* can be generically decomposed into a sum of functions $\phi_{d}^{p}$ of only *p* variables, as

$$f_{d}\left(y_{1},s\right)=\sum_{v\subseteq s:|v|=p-1}\;\phi_{d}^{p}\left(y_{1},v\right)$$ (5)

with 2 ≤ *p* ≤ *d* and where *s* denotes a multi-set of variables {{*y*_2_, …, *y_d_*}} of size *d* − 1 and *v* denotes a non-empty subset of *s* of cardinality *p* − 1. We call the minimal *p* ≥ 2 for which this decomposition is possible for all update functions *f_d_* the dynamical order *p*_dyn_ of the system. [As we are only concerned with update functions that have at least two function arguments (at least pairwise interactions), the dynamical order has to be *p*_dyn_ ≥ 2.]

Trivially, by choosing *p* = *d* we have that $\phi_{d}^{d}\left(y_{i},s\right)=f_{d}\left(y_{i},s\right)$ such that the above decomposition always exists. However, for specific functional forms *f_d_*, it can be possible to decompose higher-order interactions into a combination of *p*-ary interactions. Let us illustrate this with a concrete example. Consider the family of update functions defined for all *d* via *f_d_*(*y*_1_, …, *y_d_*) = log (*y*_1_…*y_d_*), i.e., *f*_1_ : *y*_1_ ↦ log (*y*_1_) and *f*_2_ : (*y*_1_, *y*_2_) ↦ log (*y*_1_*y*_2_). By the properties of the logarithm, we can write *f_d_* for any *d* ≥ 2 as

$$f_{d}\left(y_{1},\ldots ,y_{d}\right)=\sum_{i=2}^{d}\;[\frac{1}{d-1}\text{log}\left(y_{1}\right)+\text{log}\left(y_{i}\right)]=\sum_{i=2}^{d}\;\phi_{d}^{2}\left(y_{1},y_{i}\right)$$ (6)

and thus the dynamical order is *p*_dyn_ = 2.

From our above discussion, it should be apparent that, for a dynamical order *p*_dyn_, the dynamics on the larger hyperedges always consist of linear combinations of functions of order *p*_dyn_. Hence, one may rewrite Eq. 4 more compactly in terms of dynamics on (effective) hyperedges of size *p*_dyn_. This is akin to a higher-order equivalent of a clique expansion (*40*).

**Fig. 1.**
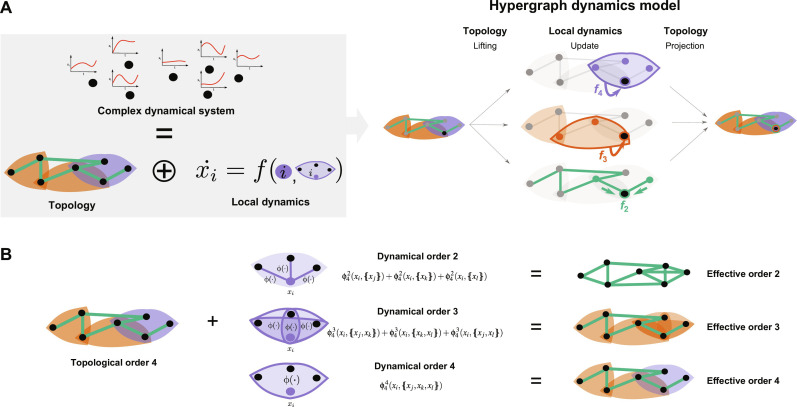
Hypergraph dynamics model and visualization of the topological, dynamical, and effective order. When modeling complex dynamical systems, it is necessary to take into account both the system’s topology and the local dynamics that are acting on the topology. We introduce a hypergraph dynamics model that separates the impact of topology and dynamics. In (**A**), we show the update process of a single node according to the full hypergraph topology. All nodes in the hypergraph are updated synchronously. In particular, the values of the nodes that are part of hyperedges of size *d* are collected. Then, an update function on these hyperedges, which defines the dynamics, is computed to obtain the update and then projected back to the node space by summing over each update. With the help of this framework, we can define and analyze different orders of the system, which we visualize in (**B**). The topological order is given by the size of the largest hyperedge in the hypergraph. To derive the dynamical order, we look at the update functions on a hyperedge of topological order 4 in more detail. In particular, the update resulting from a hyperedge of topological order 4 can consist of a linear combination of pairwise or three-way functions, which results in dynamical order 2 or 3, respectively. We can then derive the effective order of the hypergraph dynamics, which is the minimum of the topological and dynamical order. The illustration highlights that the topological order of a hypergraph is only an upper bound, not a lower bound, on the effective order of the system.

This leads us to the definition of the effective order, which combines topological and dynamical order (see Fig. 1B).

**Definition 4.** (Effective order) Consider a hypergraph dynamical system that consists of a hypergraph *H* with topological order *k* and a dynamical system {*f_d_*} with dynamical order *p*_dyn_. The effective order *p*_min_ of the system is the minimum order such that the system can be rewritten as a hypergraph dynamical system on a hypergraph *H*′ with topological order *p*_min_ and a dynamical system {*f*′*_d_*} of order *p*_min_. It is bounded by

$$p_{\text{min}}\leq \text{min}\left(k,p_{\text{dyn}}\right)$$ (7)

Note that, in most cases, we have that *p*_min_ = min (*k*, *p*_dyn_). Specifically, if the (minimal) order of the interaction functions *f_d_* is strictly increasing with *d*, then we will have equality. However, it is not difficult to construct examples where the effective order is smaller than the minimum of the dynamical and the topological order, if, for example, the interaction functions decompose in a different way when acting on an even or odd number of arguments or if we allow for different interaction functions *f_d_* for each hyperedge cardinality *d*. For a more detailed discussion, see section S2.

If *k* = *p*_dyn_, then the dynamics on none of the hyperedges decompose and the effective order of the system is given by the topological order. Consequently, we cannot reduce the hypergraph dynamical system any further. This was the case in Eq.1. Thus, the effective order of this hypergraph Kuramoto model is *p*_min_ = *k*.

In contrast, if *p*_dyn_ < *k*, then the dynamics on the hyperedges larger than the dynamical order decompose as a sum of *p*_dyn_-ary functions, and the hypergraph dynamical system can be projected onto a (new, effective) hypergraph of order *p*_min_ = *p*_dyn_ < *k*. An example for an effective order of *p*_min_ = 2 is given by Eq. 2 where the update functions for *d* = 2, …, *k* are

$$f_{d}\left(y_{1},s\right)=\sum_{y_{i}\in s}\;\text{sin}\left(y_{1}-y_{i}\right)=\sum_{y_{i}\in s}\;\phi_{d}^{2}\left(y_{1},y_{i}\right)$$ (8)

This dynamics has a dynamical order of *p*_dyn_ = 2 < *k*, and the dynamics thus decompose as a sum of pairwise functions on all hyperedges so that they are effectively network dynamics. Overall, the exchange of the order of the sum and the sin function is key to make the dynamical order of Eqs. 1 and 2 differ, leading to different effective orders of the systems.

Although conceptually useful, the derivation of the effective order as described in this section requires knowledge of the analytical form of the dynamics. In the next section, we thus present a method to learn the corresponding functions by deriving a hypergraph neural network model. Using this computational model, we can learn the dynamics from data and thus, implicitly, the effective order of the system, without prior knowledge of the functional form of the dynamics.

## LEARNING LOCAL DYNAMICS AND EFFECTIVE ORDER FROM DATA

In this section, we present a method to learn the local dynamics and thus the dynamical and effective order of the system directly from data.

### Learning a hypergraph dynamical system

In our formulation of hypergraph dynamical systems, the values of the nodes of each hyperedge are transformed by a possibly nonlinear function *f_d_*, and this update is projected back onto the nodes by summing over each update. In the spirit of this framework, we introduce a neural network–based learning approach for dynamics on hypergraphs. We call our framework hypergraph dynamics graph neural network (HyDy-GNN). Apart from minor technical adaptations, which are described in more detail in Methods, the main difference between HyDy-GNN and the analytical formulation of our dynamics is that, in HyDy-GNN, we approximate the update function *f_d_* using multilayer perceptrons (MLPs). We thus do not need to specify the functional form of the dynamics a priori but can learn the dynamics entirely from observational data, as MLPs are universal function approximators that can approximate any function to arbitrary accuracy. Technical specifications of the MLPs used in our experiments can be found in section S3.

To estimate the effective (minimal) order of the dynamics, we consider HyDy-GNNs operating on MLPs with different input dimensions. For a HyDy-GNN of order *p*_model_, only MLPs with up to *p*_model_ input variables are used to model the *p*-dimensional functions $\phi_{d}^{p}$ in Definition 3. For a general multi-set *s* of size *d* − 1, we then have

fd(yi,s)≈f^d(yi,s)={MLPdd(yi,s)if pmodel=d∑v⊆s:∣v∣=pmodel−1MLPdp(yi,v)if pmodel<d (9)

We train our HyDy-GNN using datasets $X=\{(x^{\left(1\right)},{\dot{x}}^{\left(1\right)}),\ldots ,$$\left(x^{\left(S\right)},{\dot{x}}^{\left(S\right)})\right\}$ consisting of pairs $\left(x^{\left(i\right)},{\dot{x}}^{\left(i\right)}\right)$ of state vectors *x*^(*i*)^ and gradients ${\dot{x}}^{\left(i\right)}$ of the dynamics. Our objective is for our model to output the state derivative vector $\dot{x}$ , when the state vector *x* is given as input

$$\dot{x}\approx \hat{M}\left(x\right)=\sum_{d=2}^{k}\;\sum_{E\in {ℰ}_{d}:i\in E}\;{\hat{f}}_{d}\left(x_{i},\{\{x_{j}|j\in E,j\neq i\}\}\right)$$ (10)

where ${\hat{f}}_{d}$ describes the (estimated) update functions of our HyDy-GNN. In particular, we use empirical risk minimization with a regularized *L*_1_ loss function and fit the parameters θ_*p*_model__ of HyDy-GNN, which consist of the vector of all weights of the MLPs, by minimizing the absolute difference of the prediction outputs and the provided target values of the training set *x*

$$L\left(X,\theta_{p_{\text{model}}}\right)=\sum_{i=1}^{S}{∥\hat{M}[x^{\left(i\right)}]-{\dot{x}}^{\left(i\right)}∥}_{1}+\lambda {∥\theta_{p_{\text{model}}}∥}_{2}$$ (11)

The hyperparameter λ of the *L*_2_ penalty term is optimized through a hyperparameter search. A technical detail that we need to take care of in this context is that we must assign an arbitrary ordering to the values in the multiset *s* (and thus also in the subsets *v*). To alleviate this problem, we train the MLPs based on all possible orderings of the values in the multiset *s* (or the subsets *v*) and take the mean to obtain the approximation of the *p*-ary function $\phi_{d}^{p}$ (see Methods). This comes with a computational price: As all orderings have to be computed, the order of a system this framework can be applied to is limited.

### Finding the effective model order

Using the above outligned learning paradigm, we fit a series of HyDy-GNNs with increasing orders *p*_model_ ≤ *k* (i.e., we fit models of orders up to *k*). Note that, if the chosen model order is less than the true dynamical order of the system, then the HyDy-GNN will not fit well because the MLPs of order *p*_model_ < *p*_dyn_ cannot yield a good approximation to the nonlinear dynamics induced by the larger hyperedges. However, if the model order is equal to or greater than the dynamical order of the system, then the dynamics will be learned accurately. As we take the topological order *k* as an upper bound, the true (a priori) dynamical order of the dynamics may be larger than *p*_min_. However, if we find a model with an effective order that is smaller than the topological order, this means that we have found a model order that approximates the true dynamics sufficiently well. This may happen, e.g., if the dynamics are inherently reducible to a smaller order, i.e., their dynamical order is smaller than the topological order of the hypergraph.

Hence, to find the effective model order, we need to find the model order that approximates the dynamics sufficiently well while being as small as possible. Depending on the application considered, there are multiple possible ways to select such an order. For instance, we can select the order by manual inspection or by setting a particular approximation threshold for the approximation quality of the dynamics and choosing the smallest model order that achieves this threshold.

While this is not the main focus of our work here, we discuss in the following a simple scheme to automate this process of model selection. Specifically, we define a model-corrected performance score to select the model with the lowest order *p*_model_ that produces accurate results on a given dataset, as follows

$$\text{MC}-\text{perf}\left(p_{\text{model}}|X\right)=\text{exp}[-\frac{L\left(X,\theta_{p_{\text{model}}}\right)}{L_{\text{max}}}]\text{exp}\left(-\frac{p_{\text{model}}}{k}\right)$$ (12)

where $L_{\text{max}}={\text{max}}_{p_{\text{model}}}[L\left(X,\theta_{p_{\text{model}}}\right)]$.

We provide more details on the derivation of this model-corrected performance score in section S4.

## RESULTS

In this section, we show that we can use our framework to learn the update function on the hyperedges from data. We then show that we can use these results to infer the effective order of the hypergraph dynamical system.

### Datasets

We perform our experiments on synthetic Erdős-Rényi hypergraphs and on a real hypergraph of high school students’ contact patterns (SocioPatterns dataset). Both types of hypergraphs have topological order *k* = 4. On these hypergraphs, we simulate (higher-order variants of) four common linear and nonlinear model: Kuramoto dynamics (synchronization), susceptible-infected (SI) dynamics (epidemic spread), multi-way consensus (MCM) dynamics (opinion dynamics), and linear diffusion. For each of these four dynamics, we consider different variations of the dynamics, where we restrict the update functions *f_d_* to be a sum of at most *p*-ary update functions. Details on the generation of the hypergraphs and the dynamics can be found in Methods.

We look into two possible scenarios that may occur in the real world: In the first scenario (I), we observe the state vectors and their derivatives for particular time-points. In the second scenario (II), we observe a set of trajectories of the dynamics.

For each of these settings, we create a corresponding dataset that we split into a training and test set. For the setting (I), each dataset consists of 500 independently sampled random initial state vectors and the corresponding derivatives of the dynamics at that point (see the “Datasets and dynamics considered in numerical experiments” section). This data can directly be used for training of the HyDy-GNN via empirical risk minimization (see Eq. 11). For setting (II), we generate a set of 25 trajectories, whose initial state vectors are uniformly sampled (for details see the “Datasets and dynamics considered in numerical experiments” section). Starting from the random initial conditions, we simulate 100 time steps with a time delta of Δ = 0.01 via a simple forward Euler scheme. From these trajectories, we then extract a training sequence for our model, by approximating the time derivatives $\dot{x}$ via a simple forward time difference at each time point of the trajectories. We thus create a training set of 2500 tuples of state vectors and associated derivatives that we can use to train our model. We remark that, in contrast to scenario (I), the training samples in scenario (II) are not independent, as they are coupled over time via the governing equations of the dynamics. To differentiate between the two scenarios, we will refer to the training data in the first setting (I) as point-based training data and the training data in the second setting (II) as trajectory-based training data.

### Numerical experiments: Learning the update functions

As a first step, we test whether a HyDy-GNN can accurately learn the dynamics from data. To this end, we first consider all dynamics restricted to dynamical order *p*_dyn_ = 2 such that HyDy-GNN of any order should be sufficient to fit the dynamics. We then train a HyDy-GNN with order *p*_model_ ∈ {2,3,4} to see whether we can learn the update function well.

**Fig. 2.**
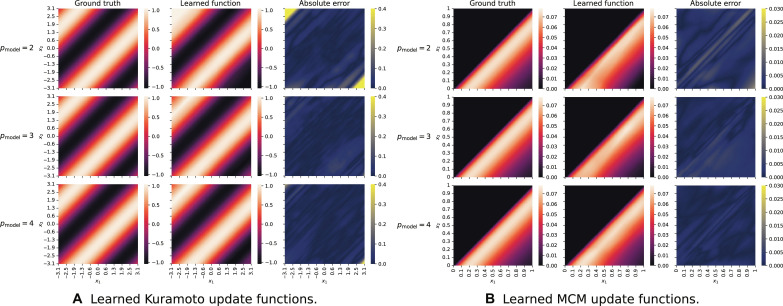
Learned update functions. Here, we see the ground-truth (left columns) and learned update functions (middle columns) resulting from a HyDy-GNN of order *p*_model_ = {2,3,4} (top to bottom) trained on a synthetic point-based dataset of pairwise Kuramoto dynamics (**A**) and pairwise MCM dynamics (**B**) of order *p* = *p*_dyn_ = 2. In particular, the *x* and *y* values correspond to a range of two-dimensional inputs of the functions, and we plot the corresponding function value. In the case when *p*_model_ > 2, we only plot the output of the MLP2 *d* with input dimension 2. The ground-truth update functions are shown in the left, and the absolute errors of the approximations, which are given by the absolute difference of the individual function values of the ground-truth and learned dynamics, are displayed in the right columns. We observe a very good approximation to the ground-truth dynamics for all model orders and both types of dynamics. This implies that our framework allows us to learn the update function of a dynamical system from data. As this is true for all model orders, we can additionally conclude that the framework can separate the effects of topology and dynamics, as it accurately captures the pairwise update functions even for higher model orders.

In Fig. 2, we see the true and learned update functions of a hypergraph neural network trained on a point-based training set of (A) pairwise Kuramoto dynamics (*p*_dyn_ = 2) and (B) pairwise MCM dynamics (*p*_dyn_ = 2).

We plot the true update functions as a function of the first and second input variables in the left columns. We show the learned dynamics for our HyDy-GNNs with model orders *p*_model_ ∈ {2,3,4} (from top to bottom) in the middle columns. When *p*_model_ > 2, we plot the output of the ${\text{MLP}}_{d}^{2}$ with input dimension 2 for simplicity. The absolute error of the approximation, i.e., the absolute difference of the individual function values of the ground-truth and learned dynamics, is shown in the right columns in Fig. 2 (A and B). As expected, we observe a good approximation of the ground-truth dynamics for all HyDy-GNN orders irrespective of the types of dynamics. In fig. S1, we show that, for other dynamics, we obtain equally good results.

### Numerical experiments: Learning the hypergraph dynamical system

#### Synthetic data

We now demonstrate how we can use our framework to learn the effective order of an observed dynamics. Specifically, we examine hypergraph dynamical systems with Kuramoto, SI, MCM, and diffusion dynamics defined in terms of *p*-ary update functions with *p* ∈ {2,3,4} (see the “Datasets and dynamics considered in numerical experiments” section). Note that, in general, *p* does not have to be the dynamical order as we only ensure that there exists some decomposition of the dynamics into *p*-variate functions, but this decomposition does not have to be the minimal one. For the diffusion dynamics, we always have *p*_dyn_ = 2 due to linearity. However, for the other update functions chosen here, the dynamical order is always equal to the chosen decomposition, i.e., *p*_dyn = *p*_ in case of the Kuramoto, SI, and MCM dynamics, as we show in section S1. As each dataset contains at least one hyperedge of every cardinality up to 4, there is always a hyperedge of the right size that does not decompose. The effective order is thus equal to the dynamical order in all our datasets.

**Fig. 3.**
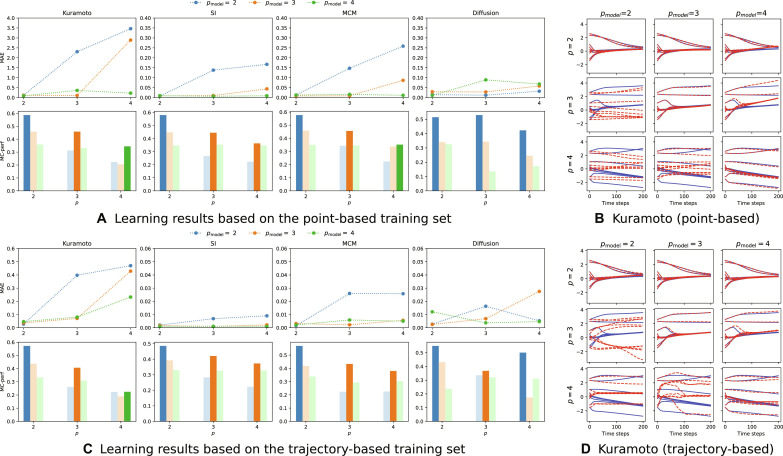
Experimental results on synthetic hypergraphs. In this figure, we compare the learning results for the point-based (**A** and **B**) and the trajectory-based (**C** and **D**) datasets and show that we are able to learn the effective order of a hypergraph dynamical system from data. The ground-truth dynamical order is *p*_min_ = *p* for Kuramoto, SI, and MCM and *p*_min_ = 2 for diffusion. In (A) in the top row, we display the mean absolute error (MAE) of cross-validation with 10 folds for all three models with *p*_model_ = {2,3,4}, which were trained on the respective dynamics datasets. In (A) in the bottom row, we plot the model-corrected performance (MC-perf), which measures accuracy accounting for model complexity. In (C), we evaluate the performance of our model for long-term trajectory predictions based on the trajectory-based training set. In (C) in the top row, we thus plot the MAE of ground-truth and a predicted trajectory. This evaluation thus includes accumulated errors. In the right column [(B) and (D)], we show an example of the long-term prediction of trajectories with 200 time steps for the two datasets. In particular, we plot ground-truth Kuramoto trajectories (blue) with *p* ∈ {2,3,4} (top to bottom) and the predicted trajectories given by a HyDy-GNN with *p*_model_ ∈ {2,3,4} (left to right).

The results of the experiments on synthetic datasets are shown in Fig. 3, where we compare the learning results for the point-based dataset in Fig. 3 (A and B) and the trajectory-based dataset in Fig. 3 (C and D). While we always trained the model with the pointwise *L*_1_ loss, as discussed in the “Learning a hypergraph dynamical system” section, we evaluate the performance with two measures: (Fig. 3A, top row) pointwise mean absolute error (MAE) and (Fig. 3C, top row) trajectory MAE (see section S5 for details). Both MAEs are calculated on a cross-validation with 10 folds. Our results show that we are able to learn the effective order from data.

In Fig. 3A (top row), we show the performance of the different models with *p*_model_ ∈ {2,3,4}, which is given by the lowest MAE of a cross-validation with 10 folds. We see in Fig. 3A (top row) that the models, whose order is equal or larger than the effective order of the hypergraph dynamical system, clearly outperform the models with an order smaller than the effective order. As the dynamical order of the diffusion dynamics is *p*_dyn_ = 2 for all hypergraph diffusion systems, all models can adequately approximate the dynamics in this case. We plot the model corrected performance in Fig. 3A (bottom row). We see that our performance score almost always selects the model with *p*_model_ = *p*_min_, which demonstrates that our method is able to infer the effective order for the synthetic training dataset. In the case of SI dynamics, we find that the observed dynamics is also approximated well by an order 3 model, which results in the selection of *p*_min_ = 3 as effective order in this particular case.

In Fig. 3C, we evaluate the performance of our model for long-term trajectory predictions based on the trajectory-based dataset. In Fig. 3C (top row), we thus plot the trajectory MAE of ground-truth and predicted trajectories. We see that the results are similar to that in Fig. 3A (top row). However, in this case, the model-corrected performance score is calculated by substituting *L*_MAE_ into Eq. 12, as described in section S5. The results also suggests the selection of an effective order of *p*_min_ = 3 for MCM and SI. An evaluation using the pointwise MAE that leads to similar results can be found in fig. S2. For SI, the model-corrected performance predicts an effective order of 3 even for the case where *p* = 4, which is not the true effective order (see section S1). This is because the impact of each multiplicand decreases with the order of the dynamics, and, thus, the dynamics can be approximated well by a lower-order model although the true effective order is higher.

In Fig. 3 (B and D), we show an example of the long-term prediction of trajectories with 200 time steps for the two datasets. Long-term prediction here means that we provide one initial condition and integrate over 200 time steps with a time delta of Δ = 0.01 via a forward Euler scheme. In particular, we plot ground-truth Kuramoto trajectories (blue) with *p*_dyn_ ∈ {2,3,4} (top to bottom) and the predicted trajectories given by a HyDy-GNN with *p*_model_ ∈ {2,3,4} (left to right).

We find that only a HyDy-GNN with large enough order approximates the long-term behavior of the dynamics well, as indicated also by the results in (A) and (C). Although the trajectory-based training set derived from a smaller amount of different hypergraph topologies (only 25 different hypergraphs are considered instead of 500 in the point-based dataset), it provides a better approximation of the trajectories [e.g., compare (B) and (D) for *p*_model_ = 4, *p* = 3]. This is because the trajectory-based dataset is biased toward the long-term behavior of the dynamics. As a result, it is more suitable for this type of long-term trajectory prediction. Generally, the results confirm that our method is capable of learning the effective order, both for the point-based and trajectory-based training sets, and the resulting models can capture the long-term behavior of the system.

#### Real-word datasets

**Fig. 4.**
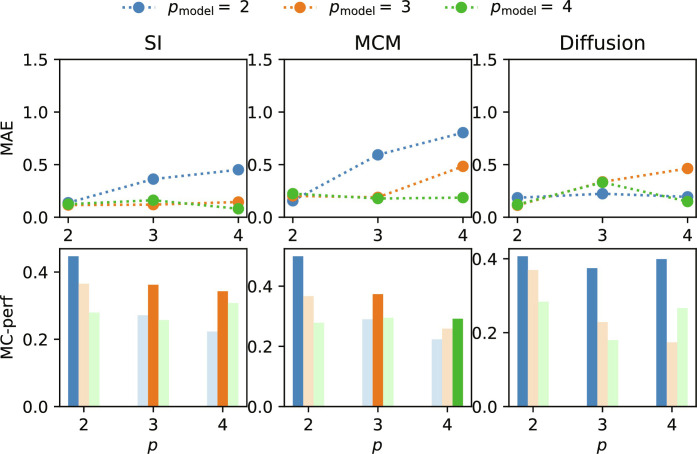
Experimental results on a real-world contact pattern hypergraph. In this figure, we display the results of experiments on three real-world datasets. In particular, we simulate epidemic spread (SI), opinion dynamics (MCM), and diffusion with order *p* ∈ {2,3,4} as all of these dynamics can be expected to appear on a contact pattern hypergraph. In the top row, we again show the MAE of cross-validation with 10 folds formodels with *p*_model_ ∈ {2,3,4}, which are trained on point-based datasets. As in the case of the synthetic datasets, we can see that, only if the model order is larger or equal to the effective order, the trajectories can be learned by the hypergraph neural networks. This is undermined by the results of the model-corrected performance in the bottom row. We see that the model order of the best model corresponds to the effective order of the system. Again, only in the case of SI dynamics, we can see that *p*_dyn_ = 4 can already be roughly captured by a model with *p*_model_ = 3. Overall, the results highlight the importance of carefully considering the type of dynamics when working with real-world hypergraph structures.

Similar to the results on the synthetic datasets, the results in Fig. 4 show that, using our method, we can learn the effective order of dynamics unfolding also on a real-world hypergraph of high-school student contacts. Here, we consider the three interaction dynamics that would be expected on a contact dataset, epidemic spread (SI), opinion dynamics (MCM), and diffusion (e.g., diffusion of ideas or information). For diffusion, a linear dynamics, the inferred effective order is correctly inferred as *p*_min_ = 2. For nonlinear dynamics such as epidemic spread and opinion formation, the best-performing model corresponds to an effective order *p*_min_ = {2,3,4} rather than the order of the hypergraph. This highlights the importance of carefully considering the type of dynamics when working with real-world hypergraph structures. Although this will not always hold, a lower-order hypergraph will be sufficient to capture the relevant dynamics in many cases. Our method enables researchers to derive how much they can simplify their models from their specific datasets of interest.

## DISCUSSION

We presented a framework to infer a hypergraph dynamical system, trading off topological and dynamical complexity. We used this framework to derive an effective hypergraph dynamical system for a given dynamics both analytically and in a data-driven way, based on observational data. In particular, using a neural network as a flexible way to approximate the local interaction dynamics, we were able to accurately learn the hypergraph dynamics and reduce the hypergraph order while respecting the observed dynamics. In this context, finding an effective dynamical order that is smaller than the topological order of the hypergraph indicates that we can “prune down” some hyperedges to be of smaller order as they do not play a role for the specific dynamics of interest. More specifically, the hyperedges in question are replaced by the set of all order-*p*_min_ subedges, and the dynamics are then rewritten so that they explicitly evolve on the smaller sub-edges. This is interesting from a point of view of model complexity reduction. Moreover, the learned models are capable of predicting the long-term behavior of dynamical systems.

We believe that our methodological approach has a wide range of potential applications and provides a starting point for a research question that is now somewhat understudied, namely, the trade-off between topological and dynamical complexity on higher-order domains such as hypergraphs, simplicial complexes, and cellular complexes. Future research is needed to further investigate this connection between (hypergraph) topology, dynamics, and effective order.

## METHODS

In this section, we formalize our hypergraph dynamical system model in more detail. In particular, we consider a dynamics in terms of the topological structure and the local dynamics. The topology of the system determines which components of the system interact locally. The dynamics and, specifically, the update functions on the edges determine how the components interact (update), and how these local interactions are globally combined (projection) is again determined by the topology.

### Hypergraph dynamical systems: Mathematical framework

Let *V* = {1, …, *N*} be a set of nodes in the system, represented by vertices. Let *x*(*t*) ∈ ℝ*^N^* be the vector of dynamical state variables of the nodes and *t* the time. The hypergraph topology is given by a set of hyperedges ℰ = {*E*_1_, …, *E_M_*}. For simplicity, we group the hyperedges according to their size *d* to obtain the whole hyperedge set *E* = *E*_1_ ∪ ⋯ ∪ *E_k_* where $E_{d}=\left\{E_{1}^{d},\ldots ,E_{M_{d}}^{d}\right\}$ and $E_{\alpha }^{d}$ is an arbitrary hyperedge with index α and size *d*, i.e., $|E_{\alpha }^{d}|=d$ . The topological order of the hypergraph is defined by its maximal hyperedge size *k*. In the case of networks, we have that *d* = 2 as the sets represent edges and thus only consist of two nodes $E_{\alpha }^{2}=\left\{i,j\right\}$ . For *d* > 2, the sets $E_{\alpha }^{d}=\left\{i,j,\ldots ,m\right\}$ represent hyperedges in which more than two nodes interact locally.

#### Topology

For each *d*-hyperedge $E_{\alpha }^{d}=\left\{i,j,\ldots ,k\right\}$ , we define its indicator matrix

$$S_{\alpha }^{d}=\left[\begin{array}{c} e_{i}^{⊤}\\ e_{j}^{⊤}\\ \vdots \\ e_{k}^{⊤} \end{array}\right]\in {\mathbb{R}}^{d\times N}$$ (13)

such that $S_{\alpha }^{d}x$ maps the node state vector *x* to the node state vector $x_{E_{\alpha }^{d}}$ that only contains the state variables of the nodes included in hyperedge $E_{\alpha }^{d}$ . We can then construct a (lifted) state vector by concatenating all the individual indicator vectors of the hyperedges by multiplying the original state vector *x* with a matrix *L_d_* ∈ ℝ*d* ∣*E_d_*∣ × *N* defined as

$$L_{d}={\left[{\left(S_{1}^{d}\right)}^{⊤}\ldots {\left(S_{M_{d}}^{d}\right)}^{⊤}\right]}^{⊤}$$ (14)

**Fig. 5.**
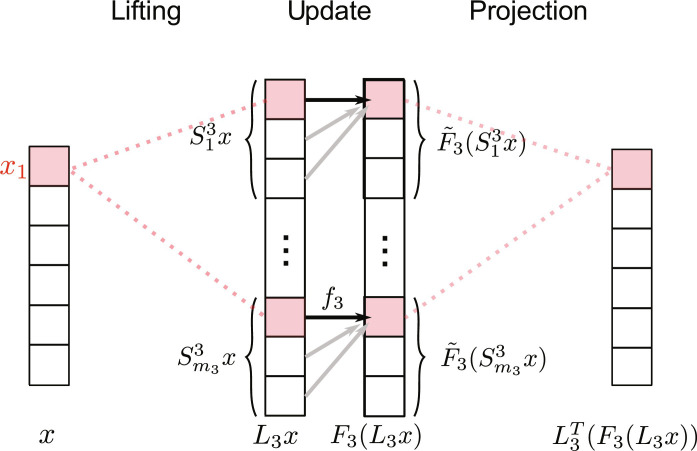
Hypergraph dynamics model. In this figure, we show the update process of a single node according to the hyperedges of order 3 in detail. Using the lifting operator *L*_3_, the values of nodes that are part of hyperedges with size of 3 are collected. Then, a possibly nonlinear function on these hyperedges is computed to obtain the update for a specific node, which is centered in these update functions. Subsequently, the obtained update values are projected back down to the node space by summing over each update.

The resulting vector corresponds to lifting the initial state vector into a higher-dimensional state space; hence, we call the matrix a lifting matrix. The lifting matrix determines which components of the system interact, represented by its nonzero entries (see Fig. 5)

#### Dynamics

The (possibly nonlinear) dynamics are then defined by the update operator *F* : ℝ*d* ∣*E_d_*∣ → ℝ*d* ∣*E_d_*∣. This operator consists of components $\tilde{F}:{\mathbb{R}}^{d}\to {\mathbb{R}}^{d}$ , which define the update for each hyperedge

$$F_{d}\left(L_{d}x\right)={\left[\begin{array}{cccc} {\tilde{F}}_{d}{\left(S_{1}^{d}x\right)}^{⊤} & {\tilde{F}}_{d}{\left(S_{2}^{d}x\right)}^{⊤} & \ldots & {\tilde{F}}_{d}{\left(S_{M_{d}}^{d}x\right)}^{⊤} \end{array}\right]}^{⊤}$$ (15)

Although the nodes are ordered, the update function is effectively a function on the interaction set and structured as

$${\tilde{F}}_{d}\left(y\right)=\left(\begin{array}{c} f_{d}\left(y_{1},\left\{\{y_{1},\ldots ,y_{d}\}\right\}\backslash y_{1}\right)\\ f_{d}\left(y_{2},\left\{\{y_{1},\ldots ,y_{d}\}\right\}\backslash y_{2}\right)\\ \vdots \\ f_{d}\left(y_{d},\left\{\{y_{1},\ldots ,y_{d}\}\right\}\backslash y_{d}\right) \end{array}\right)$$ (16)

where we recall that the individual interaction functions *f_d_* are invariant to any permutation of their last *d* − 1 arguments. In other words, the function $f_{d}\left(x_{i},\{\{x_{j}\left(t\right)|j\in E_{\alpha }^{d},j\neq i\}\}\right)$ computes the update for node *i* resulting from the hyperedge $E_{\alpha }^{d}$.

Following this generally nonlinear update step, the updates are projected back onto the nodes via the transposed lifting operator, which simply sums up the updates on all hyperedges that a node is a part of. This procedure is displayed in Fig. 5. This results in the following global dynamics

$$\dot{x}=\sum_{d=2}^{k}\;L_{d}^{⊤}F_{d}\left(L_{d}x\right)$$ (17)

For each node *i*, the dynamics can thus be written as

$$x_{i}={\left[\sum_{d=2}^{k}\;L_{d}^{⊤}F\left(L_{d}x\right)\right]}_{i}=\sum_{d=2}^{k}\;{\left\{\sum_{\alpha =1}^{M_{d}}\;[S_{\alpha }^{⊤}{\tilde{F}}_{d}(S_{\alpha }^{d}x)]\right\}}_{i}$$ (18)

=∑d=2k ∑α:i∈Eαd fd(xi,{{xj(t)∣j∈E,j≠i}}) (19)

Note that, because *F_d_* is equivariant under permutation of the ordering of the nodes in each hyperedge, the dynamics is globally equivariant under permutation of the nodes. This restricts the dynamics that are possible. For example

$$f_{d}\left(y_{1},\{\{y_{2},\cdots y_{d}\}\}\right)=\beta y_{1}+\gamma \sum_{i=1}^{d}y_{i}$$

is the only possible dynamics in this framework that can be called linear. Still, many commonly used dynamical systems can be rewritten in our framework.

### Technical details of HyDy-GNN

In our mathematical framework for hypergraph dynamical systems (Eqs. 14 to 16), the values of nodes of each hyperedge are collected by the lifting operator *L_d_* and then transformed by a possibly nonlinear function *f_d_*, and this update is projected back down to the node space by summing over each update. As already stated in the section “Learning a hypergraph dynamical system,” we adapt this framework to introduce a neural network–based learning approach for dynamics on hypergraphs. We call our hypergraph dynamics learning model HyDy-GNN.

A technical issue in this context is that the update functions *f_d_* in our mathematical framework are inviariant with respect to permutations of all but their first argument, while standard neural networks do not have this type of symmetry. To ensure the desired symmetry, we model each *f_d_* as the average over all possible permutations of the last *d* − 1 input variables. More precisely, we let *f_d_* be defined in terms of an MLP network with *p* input neurons as in Eq. 9

$${f^{\prime }}_{d}\left(y_{1},y_{2},\ldots ,y_{d}\right)=\sum_{v:|v|=p_{\text{model}}-1}\;{\text{MLP}}_{d}^{p}\left(y_{1},v\right)$$

where *v* denotes here any ordered subset of variables from the tuple (*y*_2_, …, *y_d_*), which maintain the same ordering as in the original tuple. Then, we define the symmetric neural network approximant ${\hat{f}}_{d}$ as

$$\begin{array}{c} {\hat{f}}_{d}\left(y_{1},\{\{y_{2},\ldots ,y_{d}\}\}\right)\\ =\frac{1}{\left(d-1\right)!}\sum_{\pi \in S_{d-1}}\;f\prime_{d}[y_{1},y_{\pi \left(1\right)+1},\ldots ,y_{\pi \left(d-1\right)+1}] \end{array}$$

where *S*_*d*−1_ is the symmetric group containing all the permutations of the set {1, …, *d* − 1}. We thus obtain an MLP-based function that is symmetric with respect to the last *d* − 1 variables, and we formally replace *f_d_* with ${\hat{f}}_{d}$ in Eqs. 15 and 16.

Note that this comes at a computational price. To compute the updates for each node, all *d* − 1 permutations are computed, which limits the order of the system that this framework can be applied to.

### Datasets and dynamics considered in numerical experiments

We perform our experiments on datasets of dynamics on synthetic (Erdős-Rényi hypergraphs) and real-world topologies (contact pattern dataset of high school students). We assume that the graph topology is given in the form of an adjacency list from which we extract the lifting operator. On the basis of this topology, we simulate a specific dynamics of interest.

In this work, we specifically focus on four common linear and nonlinear dynamics in network science: Kuramoto dynamics (synchronization), SI dynamics (epidemic spread), MCM (opinion dynamics), and diffusion. We define these dynamics here for general order *p* in the notation of Definition 3.

#### Synchronization (Kuramoto oscillator dynamics)

The Kuramoto model (*35*) has been applied to various synchronization phenomena of phase oscillators (*36*), ranging from power networks (*37*) to brain activity (*38*), and several works have been working on its generalization to hypergraphs (*39*). The nodal state *x_i_* corresponds to the phase of oscillator *i* and the (hyper)edges represent the couplings between the oscillators

$$\phi_{d}^{p}\left(y_{i},\left\{\{y_{1},\ldots ,y_{p}\}\right\}\backslash y_{i}\right)=\text{sin}[\sum_{j=1}^{p}\;(y_{j}-y_{i})]$$

#### Epidemic spread (susceptible-infected model)

The spreading of an infectious disease can be described by the simple SI model. The nodal state *x_i_* is equal to the infection probability of node *i*. The (hyper)edges represent infection rates between people

$$\phi_{d}^{p}\left(y_{i},\left\{\{y_{1},\ldots ,y_{p}\}\right\}\backslash y_{i}\right)=\left(1-y_{i}\right)\prod_{j=1,j\neq i}^{p}\;y_{j}$$

#### Opinion dynamics with reinforcement (MCM)

The MCM (*5*–*7*, *18*) models opinion formation with group effects. These effects are captured by a nonlinear function that scales the consensus term of the model. Depending on its form, the scaling function can capture reinforcement effects of the members of a hyperedge, and we specifically choose the model facet MCMI here, which models homophily

$$\phi_{d}^{p}\left(y_{i},\left\{\{y_{1},\ldots ,y_{p}\}\right\}\backslash y_{i}\right)=\text{exp}[\lambda \left(\frac{\sum_{j=1}^{p}\;y_{j}}{d}-y_{i}\right)]\sum_{j=1}^{p}\;\left(y_{j}-y_{i}\right)$$

In our numerical experiments, we set λ = −1.

#### Linear consensus dynamics (diffusion)

The exchange of information between autonomous agents who seek some form of cooperation can be captured by this simple consensus protocol (*41*). The links hereby describe the nodes’ influences on each other. Applications range from the spread of information in a social network to optimal control

$$\phi_{d}^{p}\left(y_{i},\left\{\{y_{1},\ldots ,y_{p}\}\right\}\backslash y_{i}\right)=\sum_{j=1}^{p}\;\left(y_{j}-y_{i}\right)$$

#### Numerical integration of dynamics

We simulate these dynamics for different, random initializations and different order *p* ∈ {2,3,4} to obtain the input/output pairs: synchronization (Kuramoto oscillator dynamics) with a uniform initialization in [−π, π], epidemic spread (SI) with a uniform initialization in [0,1], opinion dynamics (MCM) with a skewed initialization in [0,1] (as the nonlinear reinforcing effects mainly appear for skewed distributions), and spreading (diffusion) with a uniform initialization in [−1,1]. The datasets then consist of observed samples of dynamics with different orders *p* on the given hypergraph topology.

#### Synthetic and real-world hypergraphs

For our synthetically generated hypergraphs, we used a set of 500 Erdős-Rényi hypergraphs with 20 nodes. Any two edges are created with probability 0.1, any three edges with probability 0.01, and every four edges with probability 0.001. The hypergraph is generated by the xgi package (*42*) according to (*43*).

As real-world hypergraph example, we use a static version of a temporal higher-order dataset constructed from interactions recorded by wearable sensors worn by students at a high school (*44*, *45*). The hypergraph consists of 327 nodes and 7818 hyperedges, with an average size of 2.3 nodes and a maximum size of 5. However, as the dataset only contains seven edges with a size of 5, we reduced the hypergraph to consider hyperedges up to size of 4, resulting in a hypergraph of order 4. The resulting dataset includes 222 four edges, 2091 three edges, and 5498 two edges. On this hypergraph, we simulate the three social dynamics that can occur on a contact dataset: epidemic spread (SI), opinion dynamics with peer pressure (MCM), and diffusion.

## Supplementary Materials

### This PDF file includes:

Sections S1 to S5 Figs. S1 and S2
