# Supplementary material for: Learning the effective order of a hypergraph dynamical system
Source: Sci Adv. 2024 May 8;10(19):eadh4053. doi: 10.1126/sciadv.adh4053 (PMC11078196; doi:10.1126/sciadv.adh4053)
Supplement: Supplementary file 1 — Sections S1 to S5 Figs. S1 and S2 [file sciadv.adh4053_sm.pdf]

Science Advances
AAAS

Supplementary Materials for

Learning the effective order of a hypergraph dynamical system

Leonie Neuhäuser *et al.*

Corresponding author: Leonie Neuhäuser, neuhaeuser@cs.rwth-aachen.de;
Michael T. Schaub, schaub@cs.rwth-aachen.de

Sci. Adv. **10**, eadh4053 (2024)
DOI: 10.1126/sciadv.adh4053

This PDF file includes:

Sections S1 to S5
Figs. S1 and S2

1 Analytical derivation of dynamical order

We start by showing that a minimum order of $p_{\min} = 2$, resulting in pairwise network dynamics, is only possible for a function with certain linear-like characteristics.

Lemma 1. *If $f(x_1, \{\!\{x_2, \ldots, x_n\}\!\})$ is decomposable into pairwise interactions:*

$$f(x_1, \{\!\{x_2, \ldots, x_n\}\!\}) = \sum_{i=2}^{n} \phi(x_1, \{\!\{x_i\}\!\}),$$

then

$$\sum_{i=2}^{n} f(x_1, \{\!\{x_i, \ldots, x_i\}\!\}) = (n-1) f(x_1, \{\!\{x_2, \ldots, x_n\}\!\}).$$

Proof. From the decomposition of f and since multisets are unordered, we have that:

$$\begin{aligned}\sum_{i=2}^{n} f(x_1, \{\!\{x_i, \ldots, x_i\}\!\}) &= \sum_{i=2}^{n} \sum_{j=2}^{n} \phi(x_1, \{\!\{x_i\}\!\})) \\ &= (n-1) f(x_1, \{\!\{x_2, \ldots, x_n\}\!\})\end{aligned}$$

Lemma 2. *If $f(x_1, \{\!\{x_2, x_3, x_4\}\!\})$ is decomposable into three-way interactions:*

$$\begin{aligned}f(x_1, \{\!\{x_2, x_3, x_4\}\!\}) = \phi(x_1, \{\!\{x_2, x_3\}\!\}) + \phi(x_1, \{\!\{x_3, x_4\}\!\}) \\ + \phi(x_1, \{\!\{x_2, x_4\}\!\})\end{aligned}$$

then

$$\begin{aligned}& f(x_1, \{\!\{x_2, x_3, x_3\}\!\}) + f(x_1, \{\!\{x_2, x_2, x_4\}\!\}) + f(x_1, \{\!\{x_3, x_4, x_4\}\!\}) \\ &= 2f(x_1, \{\!\{x_2, x_3, x_4\}\!\}) + \frac{1}{3}\left(\sum_{i=2}^{4} f(x_1, \{\!\{x_i, x_i, x_i\}\!\})\right)\end{aligned}$$

Proof. Since multisets are unordered, we have that:

$$f(x_1, \{\!\{x_i, x_j, x_j\}\!\}) = 2\phi(x_1, \{\!\{x_i, x_j\}\!\}) + \phi(x_1, \{\!\{x_j, x_j\}\!\})$$

From this, it follows that

$$\begin{aligned}& f(x_1, \{\!\{x_2, x_3, x_3\}\!\}) + f(x_1, \{\!\{x_2, x_2, x_4\}\!\}) + f(x_1, \{\!\{x_3, x_4, x_4\}\!\}) \\ =& 2\phi(x_1, \{\!\{x_2, x_3\}\!\}) + \phi(x_1, \{\!\{x_3, x_3\}\!\}) \\ +& 2\phi(x_1, \{\!\{x_2, x_4\}\!\}) + \phi(x_1, \{\!\{x_2, x_2\}\!\}) \\ +& 2\phi(x_1, \{\!\{x_3, x_4\}\!\}) + \phi(x_1, \{\!\{x_4, x_4\}\!\}) \\ =& 2f(x_1, \{\!\{x_2, x_3, x_4\}\!\}) + \frac{1}{3}(f(x_1, \{\!\{x_2, x_2, x_2\}\!\}) \\ +& f(x_1, \{\!\{x_3, x_3, x_3\}\!\}) + f(x_1, \{\!\{x_4, x_4, x_4\}\!\}))\end{aligned}$$

With the help of Lemma 1 and Lemma 2, we can prove that the non-linear dynamics from the main part with $p \in \{2, 3, 4\}$ are higher-order functions with a dynamical order $p_{\min} = p$.

Theorem 1. *The Kuramoto, SI and MCM update function of order 3 considered in this paper are not decomposable as functions of order 2.*

Proof. Assume f is decomposable into functions of order 2 as in Lemma 1. For $f(x_1, \{\!\{x_2, x_3\}\!\}) = \sin(x_2 - x_1 + x_3 - x_1)$, let $x_1 = 0, x_2 = 0, x_3 = \frac{\pi}{2}$. Then:

$$\begin{aligned}2f(x_1, \{\!\{x_2, x_3\}\!\}) = 2\sin(\frac{\pi}{2}) = 2 \neq 0 = \sin(0) + \sin(\frac{2\pi}{2}) \\ = f(x_1, \{\!\{x_2, x_2\}\!\}) + f(x_1, \{\!\{x_3, x_3\}\!\})\end{aligned}$$

which is a contradiction.
For $f(x_1,\{\!\{x_2,x_3\}\!\})=(1-x_1)\cdot x_2\cdot x_3$, let $x_2=1,x_1,x_3=0$. But then:

$$\begin{aligned}2f(x_1,\{\!\{x_2,x_3\}\!\}) &= 2\cdot 1\cdot 1\cdot 0 = 0 \neq 1 = 1\cdot 1\cdot 1 + 1\cdot 0\cdot 0\\ &= f(x_1,\{\!\{x_2,x_2\}\!\}) + f(x_1,\{\!\{x_3,x_3\}\!\})\end{aligned}$$

which is a contradiction.
For $f(x_1,\{\!\{x_2,x_3\}\!\})=\exp(\lambda(\frac{x_1+x_2+x_3}{3}-x_1))(x_1-x_2+x_1-x_3)$, let $x_1=0,x_2=1,x_3=-1,\lambda=-1$. But then:

$$\begin{aligned}2f(x_1,\{\!\{x_2,x_3\}\!\}) &= 2\cdot\exp(0)\cdot 0 < \exp(-\frac{2}{3})\cdot -2+\exp(\frac{2}{3})\cdot 2\\ &= f(x_1,\{\!\{x_2,x_2\}\!\}) + f(x_1,\{\!\{x_3,x_3\}\!\})\end{aligned}$$

which is a contradiction.

Theorem 2. *The Kuramoto, SI and MCM update function of order* 4 *are not decomposable as functions of order* 2.

Proof. Assume f is decomposable into functions of order 2 as in Lemma 1. For $f(x_1,\{\!\{x_2,x_3,x_4\}\!\})=\sin(x_2-x_1+x_3-x_1+x_4-x_1)$, let $x_1=\frac{\pi}{2},x_2=\frac{\pi}{2},x_3=\frac{\pi}{4}$ and $x_4=-\frac{\pi}{4}$. But then:

$$\begin{aligned}&3f(x_1,\{\!\{x_2,x_3,x_4\}\!\}) = 3\sin(-\pi) = 0 \neq \sqrt{2}\\ &= \sin(0)+\sin(-\frac{3\pi}{4})+\sin(-\frac{9\pi}{4})\\ &= f(x_1,\{\!\{x_2,x_2,x_2\}\!\})+f(x_1,\{\!\{x_3,x_3,x_3\}\!\})+f(x_1,\{\!\{x_4,x_4,x_4\}\!\})\end{aligned}$$

which is a contradiction.
For $f(x_1,\{\!\{x_2,x_3,x_4\}\!\})=(1-x_1)\cdot x_2\cdot x_3\cdot x_4$, let $x_2=1,x_1,x_3,x_4=0$. But then:

$$\begin{aligned}&3f(x_1,\{\!\{x_2,x_3,x_4\}\!\}) = 3\cdot 1\cdot 1\cdot 0\cdot 0 = 0 \neq 1\\ &= 1\cdot 1\cdot 1\cdot 1 + 1\cdot 0\cdot 0\cdot 0 + 1\cdot 0\cdot 0\cdot 0\\ &= f(x_1,\{\!\{x_2,x_2,x_2\}\!\})+f(x_1,\{\!\{x_3,x_3,x_3\}\!\})+f(x_1,\{\!\{x_4,x_4,x_4\}\!\})\end{aligned}$$

which is a contradiction.
For $f(x_1,\{\!\{x_2,x_3,x_4\}\!\})=\exp(\lambda(\frac{x_1+x_2+x_3+x_4}{4}-x_1))(x_1-x_2+x_1-x_3+x_1-x_4)$, let $x_1=0,x_2=1,x_3=-1,x_4=0,\lambda=-1$. But then:

$$\begin{aligned}&3f(x_1,\{\!\{x_2,x_3,x_4\}\!\}) = 2\cdot\exp(0)\cdot 0\\ &< \exp(-\frac{3}{4})\cdot(-3)+\exp(\frac{3}{4})\cdot 3+0\\ &= f(x_1,\{\!\{x_2,x_2,x_2\}\!\})+f(x_1,\{\!\{x_3,x_3,x_3\}\!\})+f(x_1,\{\!\{x_4,x_4,x_4\}\!\})\end{aligned}$$

which is a contradiction.

Theorem 3. *The Kuramoto, SI and MCM update function of order* 4 *defined in the main part are not decomposable as functions of order* 3.

Proof. Assume f is decomposable into functions of order 3 as in Lemma 2. For $f(x_1,\{\!\{x_2,x_3,x_4\}\!\})=\sin(x_2-x_1+x_3-x_1+x_4-x_1)$, let $x_1=\frac{\pi}{2},x_2=\frac{\pi}{2},x_3=\frac{\pi}{4}$ and $x_4=-\frac{\pi}{4}$. But then:

$$\begin{aligned}&2\sin(-\pi)+\frac{1}{3}(\sin(0)+\sin(-\frac{3\pi}{4})+\sin(-\frac{9\pi}{4})) = \frac{\sqrt{2}}{3} \neq 1\\ &= \sin(-\frac{\pi}{2})+\sin(-\frac{3\pi}{4})+\sin(-\frac{7\pi}{4})\end{aligned}$$

which is a contradiction.

For $f(x_1, \{\!\{x_2,x_3,x_4\}\!\}) = (1-x_1)\cdot x_2\cdot x_3\cdot x_4$, let $x_2,x_3 = 1, x_1,x_4 = 0$. But then:

$$\begin{aligned} &2(1-0)\cdot 1\cdot 1\cdot 0 + \frac{1}{3}(1+1+0) = \frac{2}{3} \neq 1 \\ &= (1-0)\cdot 1\cdot 1\cdot 1 + (1-0)\cdot 1\cdot 1\cdot 0 + (1-0)\cdot 1\cdot 0\cdot 0 \end{aligned}$$

which is a contradiction.
For $f(x_1, \{\!\{x_2,x_3,x_4\}\!\}) = \exp(\lambda(\frac{x_1+x_2+x_3+x_4}{4} - x_1))(x_1-x_2+x_1-x_3+x_1-x_4)$, let $x_1 = 0, x_2 = 1, x_3 = -1, x_4 = 0, \lambda = -1$. But then:

$$\begin{aligned} &2\cdot\exp(0)\cdot 0 + \frac{1}{3}(\exp(-\frac{3}{4})\cdot(-3) + \exp(\frac{3}{4})\cdot 3 + 0) \\ &= -\exp(-\frac{3}{4}) + \exp(\frac{3}{4}) > 2\cdot(\exp(\frac{1}{4}) - \exp(-\frac{1}{2})) \\ &= \exp(\frac{1}{4})\cdot 1 + \exp(-\frac{1}{2})\cdot(-2) + \exp(\frac{1}{4})\cdot 1 \end{aligned}$$

which is a contradiction.

2 Examples for smaller effective orders

Note that in most cases, we have that $p_{\text{min}} = \min(k, p_{\text{dyn}})$. However, we can construct examples where the effective order is indeed smaller than the minimum of the dynamical and the topological order. Consider the family of update functions defined for $d \leq 3$ via $f_d(y_1,\ldots,y_d) = \sin(y_1\ldots y_d)$, i.e., $f_1 : y_1 \mapsto \sin(y_1)$, $f_2 : (y_1,y_2) \mapsto \sin(y_1y_2)$, and $f_3 : (y_1,y_2,y_3) \mapsto \sin(y_1y_2y_3)$. For $d > 3$, the function decomposes into a sum of pairwise couplings, i.e. $f_d(y_1,\ldots,y_d) = \sum_{i=1}^{d} \sin(y_1y_i)$. This dynamics clearly has a dynamical order of $p_{\text{dyn}} = 3$. Now consider two different hypergraphs of topological order $k = 4$, H_1 and H_2, whereas H_1 only exists of one hyperedge of size 4 and H_2 additionally contains hyperedges of size 3.

If we observe the constructed dynamics on H_1, we would derive an effective order $p_{\text{min}} = 2$ as the dynamics on the size 4 hyperedge would decompose into a sum of pairwise couplings. Therefore, the effective order would be indeed smaller than the minimum of topological and dynamical order. However, if the dynamics would be observed on H_2, the dynamical order of 3 would manifest on the hyperedges of size 3, which are present in this hypergraph topology.

A practical example for such a type of dynamics would be a group dynamics where the group effect breaks down if groups are larger than a certain size. In that case, the dynamics in larger groups is more realistically given by pairwise couplings as otherwise we would assume that all possible subgroups of one order lower exist.

3 Technical specifications of the MLPs

In our experiments we used MLPs with ReLu activation functions with n layers and layer size $2^n * 16$. The number of layers n was chosen through a hyperparameter search, varying $n \in \{1,2,3\}$. For further details, we refer to the released code associated with the paper.

4 Model-corrected performance score

As discussed in the main part, we define a model-corrected performance score which allows us to select the model with the lowest model order p_{model} that also produces accurate results on a given dataset. Having trained a series of models with $p_{\text{model}} \in \{2,\ldots,p_{\text{max}}\}$ on a given dataset, we have a set of minimal L_1 loss function values $L(x,\theta_{p_{\text{model}}})$, where x is the training set and $\theta_{p_{\text{model}}}$ the parameters of a model with order p_{model}, given by the weights of the MLPs. The optimal model that takes both factors into account should minimise both the L_1 loss function $L(x,\theta_{p_{\text{model}}})$ and a penalty term $P(p_{\text{model}})$ that accounts for the complexity of the model, similar to a regularisation term. Here, we choose $P(p_{\text{model}}) = p_{\text{model}}$ and normalise both terms in order to operate on the same scale. By choosing p_{model} such that the sum of the loss function and the penalty term is minimised, we can derive an estimate of the effective order $\hat{p}_{\text{min}}$:

$$\hat{p}_{\text{min}} = \underset{p_{\text{model}}}{\arg\min} \frac{L(x,\theta_{p_{\text{model}}})}{L_{\text{max}}} + \frac{p_{\text{model}}}{k} \tag{1}$$

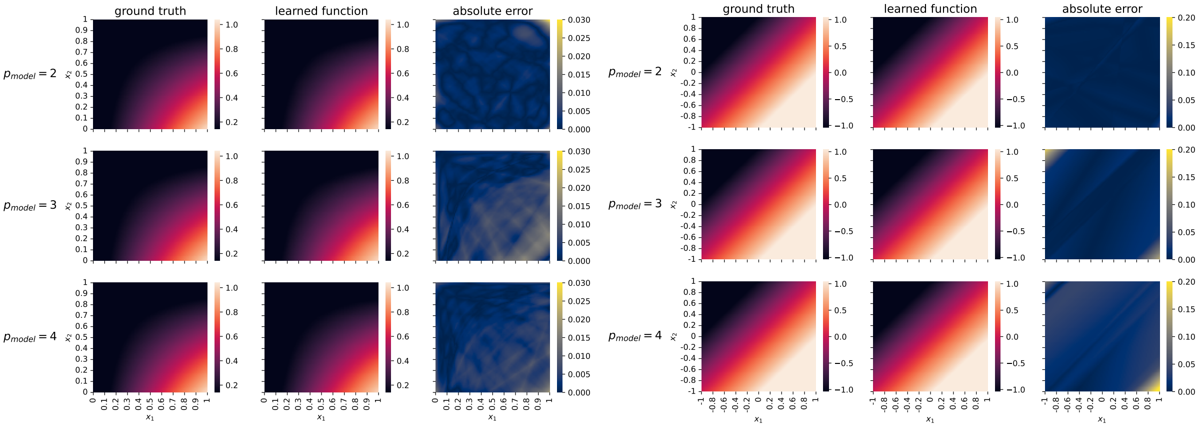

(a) Learned SI update functions.

(b) Learned diffusion update functions.

Figure S1. Learned and ground truth update functions of the dynamics. In these two examples, we see the learned update functions (left column) of a Hypergraph Neural Network which was trained on a dataset of pairwise SI and Diffusion dynamics, i.e. $p_{\text{dyn}} = 2$ (centre column). The hypergraph topology is given by Erdős-Réyni hypergraphs with topological order $k = 4$. The training was done for HyDy-GNNs of order $p_{\text{model}} \in \{2, 3, 4\}$ (top to bottom). The absolute error is displayed in the right column. We observe a very good approximation of the ground truth dynamics for all model orders. This implies that our framework enables us to learn the update function of a dynamical system from data. As this is true for all model orders we can additionally conclude that the framework can separate the impact of topology and dynamics, as it precisely captures the pairwise update functions, even for higher model orders.

where $L_{\max} = \arg\max_{p_{\text{model}}} \left(L(x, \theta_{p_{\text{model}}}) \right)$. Rewriting this term we get:

$$\hat{p}_{\min} = \arg\min_{p_{\text{model}}} \frac{L(x, \theta_{p_{\text{model}}})}{L_{\max}} + \frac{p_{\text{model}}}{k} \tag{2}$$

$$= \arg\max_{p_{\text{model}}} - \frac{L(x, \theta_{p_{\text{model}}})}{L_{\max}} - \frac{p_{\text{model}}}{k} \tag{3}$$

$$= \arg\max_{p_{\text{model}}} \exp\left(- \frac{L(x, \theta_{p_{\text{model}}})}{L_{\max}} - \frac{p_{\text{model}}}{k} \right) \tag{4}$$

$$= \arg\max_{p_{\text{model}}} \exp\left(- \frac{L(x, \theta_{p_{\text{model}}})}{L_{\max}} \right) \exp\left(- \frac{p_{\text{model}}}{k} \right). \tag{5}$$

We thus derive our model-corrected performance score:

$$\text{MC-perf}(p_{\text{model}}|x) = \exp\left(- \frac{L(x, \theta_{p_{\text{model}}})}{L_{\max}} \right) \exp\left(- \frac{p_{\text{model}}}{k} \right)$$

where $L_{\max} = \arg\max_{p_{\text{model}}} \left(L(x, \theta_{p_{\text{model}}}) \right)$.

Taking a probabilistic perspective, we have that the normalised model-corrected performance score can be interpreted as a probability distribution (as it is additionally positive due to the exponential function). The effective order $\hat{p_{\text{model}}}$ is thus derived by implicitly maximising the conditional probability $\mathbb{P}(\theta|x)$ of the model parameters θ being optimal given the observations x:

$$\begin{aligned} \arg\max_{p_{\text{model}}} (\text{MC-perf}(\theta|x)) &\approx \arg\max_{\theta} (\mathbb{P}(x|\theta)\mathbb{P}(\theta)) \\ &= \arg\max_{\theta} \left(\frac{\mathbb{P}(x|\theta)}{\mathbb{P}(x)} \mathbb{P}(\theta) \right) \\ &= \arg\max_{\theta} (\mathbb{P}(\theta|x)) \end{aligned}$$

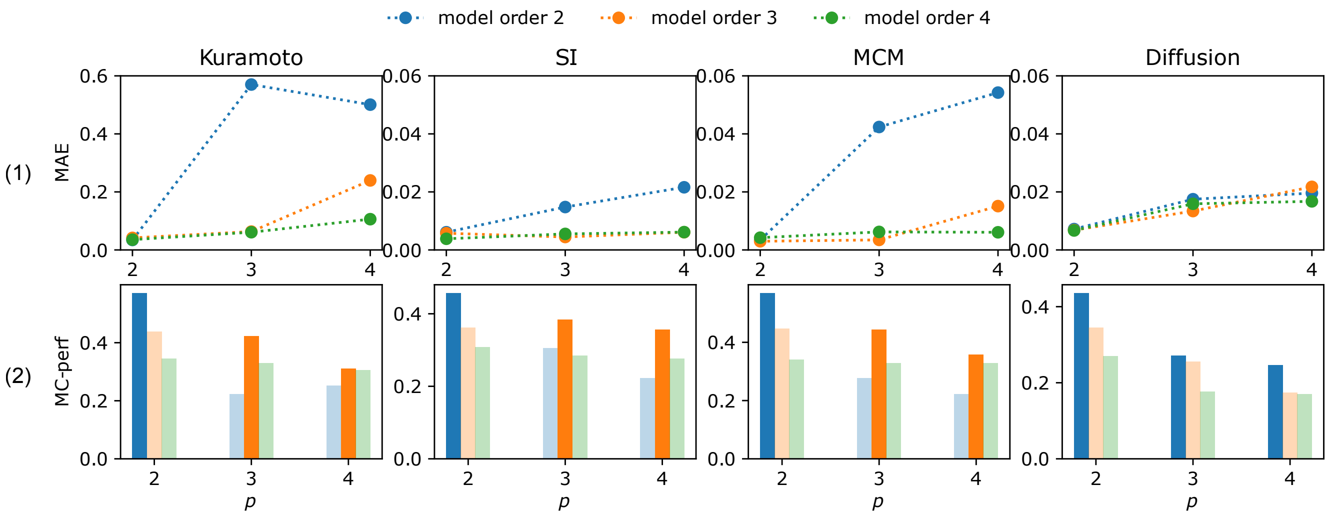

Figure S2. **Evaluation of trajectory dataset without accumulated errors.** We evaluate the performance of our model for long-term trajectory predictions based on the trajectory training set. We now use the original evaluation score $L(\mathscr{X}, \theta_{p\text{model}})$, not accumulating errors over the whole trajectory, We see that the results are similar to Figure 3 in the main text, whereas in this case the model-corrected performance score also suggests $p_{\min} = 3$ for a dynamics with $p_{\text{dyn}} = 4$ for Kuramoto, even though the model of order 4 leads to a better results.

5 Trajectory MAE

Given a set of trajectories $\mathscr{T}$, in which each trajectory $e \in \mathscr{T}$ results in a discrete set of training samples $\{x_e^{(1)}, \dots, x_e^{(n)}\}$, the MAE over a whole trajectory is given as $L_{\text{traj}}(\mathscr{X}, \theta_{p_{\text{model}}}) = \frac{1}{|\mathscr{T}|} \sum_{e \in \mathscr{T}} \frac{1}{n} \sum_{i=1}^{n} \|\hat{M}(x_e^{(i)}) - \dot{x}_e^{(i)}\|_1$. Note that we do not use this term for training, but only for evaluation, which now includes accumulated errors. Using the trajectory MAE for model selection, we now also have

$$\begin{aligned}\text{MC-perf}_{\text{traj}}(p_{\text{model}} | \mathscr{X}) \\ = \exp\left(-\frac{L_{\text{traj}}(\mathscr{X}, \theta_{p_{\text{model}}})}{L_{\max}}\right) \exp\left(-\frac{p_{\text{model}}}{k}\right)\end{aligned} \tag{6}$$

where $L_{\max} = \max_{p_{\text{model}}} \left(L_{\text{traj}}(\mathscr{X}, \theta_{p_{\text{model}}})\right)$. An evaluation using the pointwise MAE using $L(\mathscr{X}, \theta_{p_{\text{model}}})$ can be found in fig. S2. We see that the results are very similar.
